# Critical Left Subclavian Artery Stenosis With Retrograde Vertebral Flow: A Case Report and Literature Review

**DOI:** 10.7759/cureus.51027

**Published:** 2023-12-24

**Authors:** Andreas Montano, Martin Halicek, Victor Collier, Brooke Reese

**Affiliations:** 1 Internal Medicine, Grand Strand Medical Center, Myrtle Beach, USA; 2 Diagnostic Radiology, Emory University, Atlanta, USA; 3 Medicine, Edward Via College of Osteopathic Medicine, Spartanburg, USA

**Keywords:** vertebral artery, balloon angioplasty with stent, subclavian artery, subclavian steal syndrome, subclavian artery stenosis, subclavian artery occlusion

## Abstract

Subclavian artery stenosis is a rare condition associated with significant morbidity and mortality, making prompt recognition and treatment essential. We present a case of left-sided subclavian artery occlusion with neurological symptoms, including vertigo, unsteady gait, and left upper extremity pain and paresthesia. The patient’s symptoms had been progressing over several months. Her risk factors included age, hyperlipidemia, and poorly controlled blood pressure with resultant arteriosclerosis throughout her vasculature. An arteriogram demonstrated critical stenosis of the left subclavian with retrograde flow through the left vertebral artery. Aspirin and clopidogrel were initiated prior to successful balloon angioplasty and stenting. After stent placement, the patient had minimal residual subclavian stenosis and anterograde vertebral artery flow. In this case report, we discuss clinical presentation, typical examination and imaging findings, and treatment options for subclavian stenosis including medical management and revascularization procedures.

## Introduction

Subclavian artery occlusion most commonly occurs on the left side and presents with neurological and left upper extremity symptoms. The left subclavian artery originates from the aortic arch and gives rise to the left vertebral artery and the vascular supply of the left upper extremity. High-grade stenosis of the left subclavian near the take-off point from the aorta causes posterior cerebellar symptoms, such as vertigo, dizziness, and vision changes, due to retrograde flow through the left vertebral artery in addition to left extremity pain and numbness due to limited blood supply. Treatment options consist of atherosclerotic risk factor management, antiplatelet therapy, percutaneous transluminal balloon angioplasty, and vessel bypass surgery.

## Case presentation

An 89-year-old female with a history of hyperlipidemia and untreated hypertension presented to our facility after an outpatient computed tomography angiography (CTA) of the chest and neck showed critical occlusion of the left subclavian artery along with calcifications of the aortic arch and descending aorta.

The patient had presented to her primary care physician one week prior with several weeks of progressive vertigo, gait instability, falls, blurry vision, and numbness/tingling in her left arm. Symptoms were intermittent in nature without any clear relieving or exacerbating factors. Physical examination was remarkable for a left radial pulse that was diminished compared to the right side, bruits over the bilateral carotids and anterior chest, and equal brachial blood pressures.

**Figure 1:**
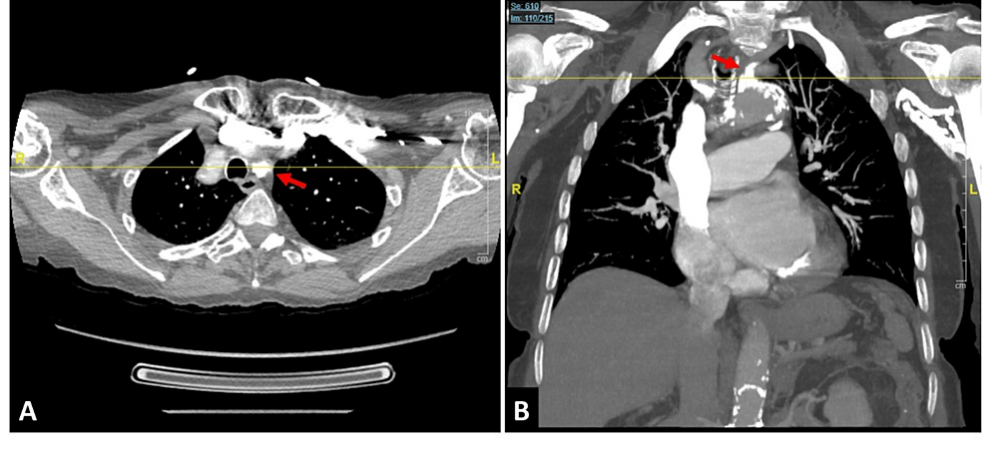
CTA chest. High-grade short-segment stenosis of origin of left subclavian artery (red arrow), near the take-off point from the aorta in axial (A) and coronal (B) views. Of note, post-stenotic segments of the left subclavian are patent. CTA, computed tomography angiography.

Repeat computed tomography angiography (CTA) of the chest, head, and neck demonstrated high-grade short-segment stenosis at the origin of the left subclavian artery, near the aortic take-off point, with post-stenotic segments patent (Figure 1).

**Figure 2:**
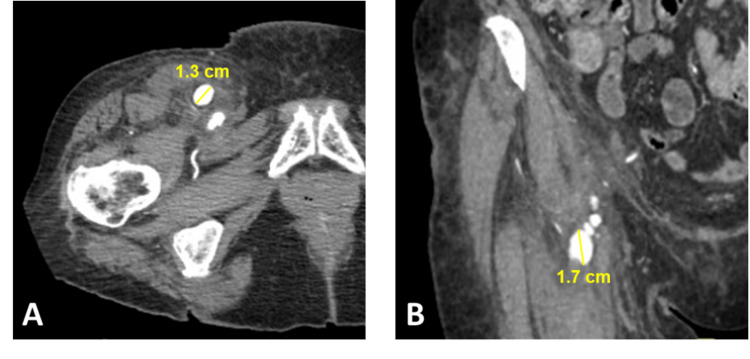
CTA abdomen/pelvis. The day following the initial interventional procedure, CTA was obtained demonstrating symptomatic pseudoaneurysm arising from the right common femoral artery with multiple lobulations, greatest dimensions noted in axial (A) and coronal (B) views. Adjacent soft-tissue edema noted. CTA, computed tomography angiography.

Vascular surgery was consulted and recommended angiogram with interventional radiology for further evaluation and possible intervention. Using a right common femoral artery access site, an angiogram revealed critical left subclavian artery origin stenosis. Extensive attempts at angioplasty were unsuccessful. The procedure was complicated by an iatrogenic femoral pseudo-aneurysm requiring thrombin injection (Figure 2).

Angioplasty was reattempted using real-time sonographic guidance and a left common femoral artery access site. Angiogram re-demonstrated critical stenosis of the origin of the left subclavian artery with retrograde flow in the left vertebral artery. Using careful coaxial technique, guidewires, and catheters, the critical stenosis was eventually crossed and pre-dilated with a 4 mm balloon. It was then dilated with a 6 mm balloon and stented with a 6 x 27 mm stent.

**Figure 3:**
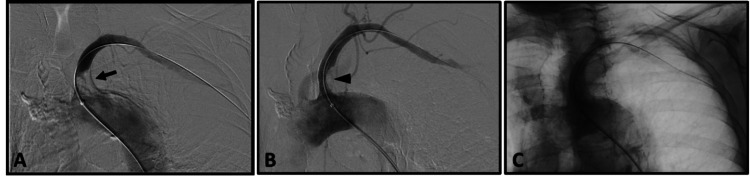
Subclavian arteriography. (A) Critical stenosis of the origin (black arrow) of the left subclavian artery appears patent. Retrograde flow in the left vertebral artery was demonstrated (not shown). (B) Successful pre-dilation and subsequent balloon stenting with a 6 x 27 mm stent (black arrowhead). (C) The completion arteriogram demonstrated only minimal residual stenosis of the subclavian artery, and there is now antegrade flow in the left vertebral artery.

The completion arteriogram demonstrated only minimal residual stenosis, with persistent post-stenotic dilatation of the proximal-most subclavian artery (Figure 3). Antegrade flow in the left vertebral artery was achieved following successful intervention.

The patient was observed for two additional days and determined to be medically stable. She was discharged on dual anti-platelet therapy with aspirin and clopidogrel and high-intensity statin, with vascular surgery and primary care follow-up.

## Discussion

Subclavian artery stenosis (SAS) affects up to 2% of the whole population [1] and is associated with significant morbidity and mortality [2]. Atherosclerosis is the most common cause, accounting for up to 80% of cases [3,4]. Risk factors for atherosclerosis include hypertension, hyperlipidemia, smoking, diabetes, and advanced age. Other less common causes include familial hypercholesterolemia, fibromuscular dysplasia, Takayasu arteritis, neurofibromatosis, and inflammation due to radiation exposure [3]. 

Left-sided subclavian artery stenosis is more common than right-sided SAS, with a reported prevalence of 70-80% of all cases of SAS [5]. Most patients will remain asymptomatic [6] due to incomplete stenosis or revascularization by retrograde flow through the vertebral artery. However, critical stenosis can lead to symptoms such as arm claudication and weakness, with decreased pulses and blood pressure in the affected arm. When a patient with a subclavian artery occlusion becomes symptomatic, vertigo is the most frequent symptom. A study of 168 patients suffering from subclavian steal syndrome revealed that vertigo was present in 52% of cases and tinnitus in 4% [7]. Other common clinical symptoms are neurologic, which are experienced by about 5% of patients, including dizziness, vertigo, and falls [8]. On physical examination, the patients can have a systolic blood pressure difference of greater than 10 millimeters of mercury (mmHg) between the affected and contralateral upper extremities [3]. Due to this pressure difference, patients may also experience muscle fatigue, rest pain, and finger necrosis in the affected arm [3]. Subclavian stenosis is found in 7% of the clinical population and a difference in brachial blood pressures is a good clinical clue but is not always present [9]. Interestingly, our patient lacked the usual discrepancy in brachial systolic blood pressures typically observed in cases of subclavian stenosis, a key diagnostic indicator. 

SAS can often be misdiagnosed as cervical radiculopathy or discopathy due to symptom and presentation overlap [1]. Therefore, consideration of vascular imaging is important in patients with risk factors or exam findings consistent with SAS. Diagnosis of SAS is typically made with non-invasive imaging studies, such as arterial ultrasound, computed tomography angiography (which was the case for our patient), or magnetic resonance angiography. Invasive angiography can also be performed for diagnosis and treatment planning. 

Medical management for SAS aims to reduce the progression of atherosclerosis and cardiovascular complications. In these patients, glycemic control is necessary with the target HbA1c of less than 7% [3], high-intensity statin therapy to reduce low-density lipoprotein (LDL) cholesterol, as well as an angiotensin-converting enzyme (ACE) inhibitors for blood pressure reduction if needed [1]. Additionally, current recommendations include the initiation of antiplatelet therapy for patients with SAS [5].

Current SAS management guidelines highly advocate for dual antiplatelet therapy (DAPT) after subclavian stenting, using aspirin and clopidogrel [3,5]. However, no randomized control trials have been performed to investigate single-agent versus dual-agent antiplatelet regimens [3]. Additionally, the duration of DAPT remains unclear after stenting, before switching to single-agent antiplatelet therapy for a long term.

Interventional treatment options for SAS can be endovascular, with angioplasty and stenting, or surgical revascularization with bypass grafting. With regard to interventional procedures, percutaneous transluminal angioplasty (PTA) is currently the treatment of choice for patients with subclavian artery stenosis and has been shown to achieve long-term patency [8,10]. PTA is a minimally invasive procedure that has been shown to be effective in improving symptoms and restoring blood flow in patients with subclavian artery stenosis [3,11]. There remains some debate regarding the optimal treatment approach for subclavian artery stenosis, particularly in terms of the use of medical management versus interventional procedures [6,12]. However, a recent cohort study of 100 patients demonstrated that medical management when combined with either PTA or bypass surgery improved all-cause mortality by over three-fold and lowered adverse cardiovascular outcomes, compared to medical management alone [5].

In our case report, the patient underwent successful PTA with stenting, only minimal residual subclavian stenosis, and re-establishment of antegrade flow in the left vertebral artery, and she was discharged on dual anti-platelet therapy with aspirin and clopidogrel and high-intensity statin.

## Conclusions

In conclusion, in this report, we present a classic case of critical left subclavian artery stenosis with retrograde flow through the left vertebral artery causing neurological symptoms, including vertigo and ataxia, in addition to left upper extremity claudication. An arteriogram with balloon angioplasty and stenting was successfully performed to achieve minimal residual subclavian stenosis and anterograde vertebral artery flow. Subclavian artery stenosis is a rare condition but is associated with high mortality, which can lead to a variety of symptoms and complications. Diagnosis is made with clinical history and physical examination along with imaging for cases of high clinical suspicion. Treatment options include medical management and interventional procedures, and current studies suggest that combining medical management and interventional procedures leads to optimal outcomes.
